# Treatment Strategies for Improving the Surgical Outcomes of Ruptured Abdominal Aortic Aneurysm: Single-Center Experience in Japan

**DOI:** 10.3400/avd.oa.21-00086

**Published:** 2022-03-25

**Authors:** Yasumi Maze, Toshiya Tokui, Masahiko Murakami, Teruhisa Kawaguchi, Ryosai Inoue, Bun Nakamura, Koji Hirano, Shuji Chino, Ken Nakajima, Noriyuki Kato

**Affiliations:** 1 Department of Thoracic and Cardiovascular Surgery, Ise Red Cross Hospital, Ise, Mie, Japan; 2 Department of Radiology, Ise Red Cross Hospital, Ise, Mie, Japan; 3 Department of Radiology, Mie University Hospital, Tsu, Mie, Japan

**Keywords:** ruptured abdominal aortic aneurysm, endovascular aneurysm repair, intra-aortic balloon occlusion, abdominal compartment syndrome, open abdominal management

## Abstract

**Objective**: We aimed to examine the surgical outcomes of ruptured abdominal aortic aneurysm cases at our hospital and considered strategies for improvement.

**Material and Methods**: We examined the preoperative characteristics of hospital mortality, postoperative complications, and long-term outcomes of 91 surgical cases of ruptured abdominal aortic aneurysm performed between January 2009 and December 2020 at our hospital.

**Results**: Of the 91 cases, 24 died at the hospital (mortality, 26.3%). Mortality was mostly due to hemorrhage/disseminated intravascular coagulation and intestinal necrosis. Ten patients required preoperative aortic clamp by thoracotomy or insertion of intra-aortic balloon occlusion, and eight of them died. Ten patients required open abdominal management due to abdominal compartment syndrome, and five of them died. There was no significant difference between the two groups in terms of the long-term results of the open repair and abdominal endovascular aneurysm repair (EVAR).

**Conclusion**: To improve the surgical outcomes of ruptured abdominal aortic aneurysms, it is necessary to start surgery immediately. Therefore, the choice of surgical method (open surgery or EVAR) should be based on the resources and discretion of the hospital. To prevent postoperative intestinal necrosis, risk factors for acute compartment syndrome should be considered, and open abdominal management should be introduced.

## Introduction

Stable outcomes of elective surgical treatment methods for abdominal aortic aneurysm, such as open repair and endovascular aneurysm repair (EVAR), have been obtained. However, for ruptured abdominal aortic aneurysm (rAAA), the outcomes are still poor; hence, new or improved treatment strategies are needed. Recently, EVAR has improved the surgical outcomes of rAAA.^1–5)^ Moreover, EVAR confers not only survival advantage but is also cost effective.^6)^ EVAR has been performed by radiologists at our hospital. However, until recently, we had only a few full-time radiologists. Therefore, even if EVAR was indicated, there were several cases in which EVAR could not be performed. In this study, we report the surgical outcomes of open repair and EVAR for treating rAAA at our hospital and we discuss treatment strategies for improving the surgical outcomes.

## Materials and Methods

Surgical procedures and data collection were performed at the Ise Red Cross Hospital, Ise, Japan. Clinical outcome data were obtained from the hospital’s patient records or from the patient’s family doctor. This study was approved by the Institutional Review Board of the Ise Red Cross Hospital (approval number ER2021-23), and the need for informed consent was waived due to the retrospective nature of the study. All methods were performed in accordance with the relevant guidelines and regulations.

Between January 2009 and December 2020, 555 cases of abdominal aortic aneurysm surgery were performed at the Ise Red Cross Hospital. There were 371 open repair procedures, 173 EVAR procedures, 5 extra-anatomical bypass procedures, 4 cases in which surgery could not be completed due to hemodynamic disruption, and 2 cases of lumbar artery ligation due to end leak after EVAR. Of these, 91 were ruptured cases; hence, they were further examined. The choice of surgical procedure for each case was determined by a vascular surgeon who was familiar with EVAR. Since EVAR was performed by a radiologist at our hospital, even if EVAR was indicated, open repair was performed if the radiologist could not come to the hospital immediately. The surgical procedure was selected according to the patient’s general condition, operating room preparedness, and arrival time of the vascular surgeon and radiologist.

This study aimed to evaluate the in-hospital mortality and morbidity after surgery for rAAA, to compare the preoperative situation of dead and surviving cases, and to investigate the causes of death. We examined the details of cases in which open abdominal management (OAM) was performed to prevent abdominal compartment syndrome (ACS). Furthermore, we compared and examined the preoperative status, surgical outcomes, and long-term survival of both EVAR and open-repair groups.

### Definitions

Open repair was defined as abdominal aortic replacement with an artificial graft in situ. OAM was defined as a method for treating by vacuum assisted closure,^7)^ in this method, the abdomen remained open to prevent ACS. Preoperative shock was defined as a systolic blood pressure <80 mmHg. The door-to-procedure time was defined as the time from arrival at the hospital to the time of skin incision.

### Statistical analyses

All statistical analyses were performed using the statistical software EZR (Easy R) on R commander.^8)^ Continuous variables are presented as mean±standard deviation. They were compared using the Student’s t-test. Categorical variables are presented as numbers and percentages and were compared using the χ^2^ test. The Kaplan–Meier survival curves were created to assess the differences in survival between the EVAR and open-repair groups. Survival distributions were compared using the log-rank tests. Statistical significance was set at P<0.05.

## Results

Of the 91 cases examined, 67 remained alive, while 24 died at the hospital; hence, the hospital mortality rate was 26.3%. Of those who survived, 50 patients were men and 17 were women. Their mean age was 47–91 (75.3±9.8) years. The surgical procedures were open repair in 50 cases, EVAR in 13, and extra-anatomical bypass in 4. Of the 24 patients who died, 22 were men and 2 were women. Their mean age was 65–93 (75.9±7.6) years, and the surgical procedures were open repair in 20 cases. In the remaining four cases, surgery could not be completed due to hemodynamic disruption.

The preoperative conditions of the group of patients that survived and the group of those who died were compared. Preoperative shock, aortic clamp by thoracotomy, and insertion of an intra-aortic balloon occlusion (IABO) were significantly more common in patients who died. In the evaluation of the type of rupture by Fitzgerald classification,^9)^ type IV was significantly more common in patients who died. There were 16 cases of Fitzgerald classification type IV, but in 10 cases, aortic clamping by thoracotomy or insertion of IABO was required. In Fitzgerald classification type IV cases, six were non-serious hemodynamic disruption. There was no difference in door-to-procedure time between the surviving and dead cases. Moreover, no EVAR was performed among those who died (**Table 1**).

**Table 1:** Table 1 Preoperative characteristics and surgical procedures

	Surviving cases (n=67)	Dead cases (n=24)	p value
Age	75.3±9.8	75.9±7.6	0.798
Female sex	17 (25.3%)	2 (8.3%)	0.088
Preoperative shock cases	21 (31.3%)	18 (75%)	p<0.001
Preoperative requiring aortic clamp or insertion of IABO	2 (2.9%)	8 (33.3%)	p<0.001
Fitzgerald classification			
I	23 (34.3%)	4 (16.6%)	0.124
II	19 (28.3%)	4 (16.6%)	0.192
III	14 (20.8%)	4 (16.6%)	0.772
IV	6 (8.9%)	10 (41.6%)	p<0.001
Unknown	5 (7.4%)	2 (8.3%)	1.0
Door-to-procedure (h)	3.3±2.4	4.5±10.2	0.363
Surgical procedures			
Open repair	50 (74.6%)	20 (83.3%)	0.573
EVAR	13 (19.4%)	0	0.017
Others	4 (5.9%)	4 (16.7%)	0.2

IABO: intra-aortic balloon occlusion; EVAR: endovascular aneurysm repair

Therefore, when comparing the EVAR and open-repair groups, the EVAR group was significantly older, and although there was no significant difference, the patients in the open-repair group underwent more frequently preoperative shock than those in the EVAR group. There was no difference between the two groups in terms of Fitzgerald classification and door-to-procedure time (**Table 2**).

**Table 2:** Table 2 The preoperative characteristics of EVAR and open repair

	EVAR (n=13)	Open repair (n=70)	p value
Age	83.6±5.0	74.0±9.4	p<0.001
Female sex	3 (23.0%)	16 (22.8%)	1.0
Preoperative shock cases	2 (15.3%)	32 (45.7%)	0.063
Cases requiring aortic			
clamp or insertion of IABO	0	8 (11.4%)	0.346
Fitzgerald classification			
I	5 (38.4%)	20 (28.5%)	0.518
II	6 (46.1%)	15 (21.4%)	0.082
III	2 (15.3%)	15 (21.4%)	1.0
IV	0	15 (21.4%)	0.112
Unknown	0	5 (7.1%)	1.0
Door-to-procedure (h)	3.6±1.7	3.8±6.8	0.462
Hospital death	0	20 (28.5%)	0.031

IABO: intra-aortic balloon occlusion; EVAR: endovascular aneurysm repair

The causes of death were hemorrhage/disseminated intravascular coagulation (DIC) in ten cases, postoperative intestinal necrosis in nine, lower limb compartment syndrome in two, brain death in two, and acute respiratory distress syndrome in one. Ten patients required preoperative aortic clamp by thoracotomy or insertion of IABO to maintain hemodynamics, and eight of them died. Of the ten cases in which hemorrhage and DIC were the causes of death, insertion of IABO was performed in three cases and aortic clamp by thoracotomy was performed in two before surgery. In addition, ten patients required OAM to prevent ACS, and half of them died. Of the five deaths, four occurred within four days after surgery. In other words, in many cases requiring OAM, life could be saved if the patient survived in the acute phase. All patients who required OAM underwent primary OAM, i.e., there were no cases of secondary OAM after primary abdominal closure. Furthermore, intra-abdominal pressure (IAP) performed after surgery from a few years ago, and the IAP value was ≤12 mmHg in all cases.

**Figure 1:**
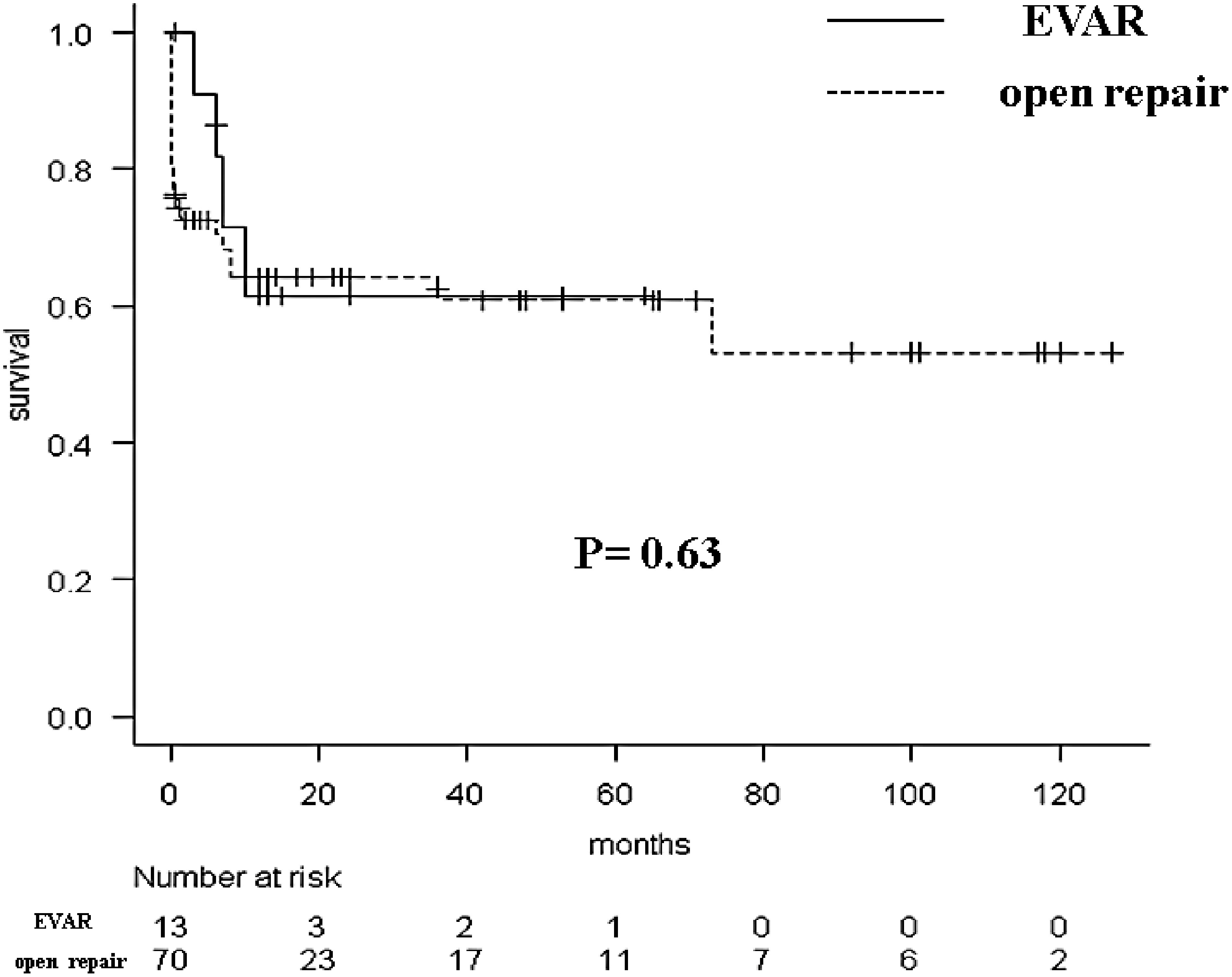
Fig. 1 The comparison of the 5-year survival between the endovascular aneurysm repair and the open-repair groups for ruptured abdominal aortic aneurysms.

The overall mean follow-up period was 22.1±33.1 (median, 6; range, 0.02–127) months. The mean follow-up period was 16.4±19.0 (median, 10; range, 0.5–64) months in the EVAR group and 24.1±35.0 (median, 5.5; range, 0.02–127) months in the open-repair group. We subsequently examined the long-term results of the open-repair and EVAR groups. The 1-year survival rate was 64.2%±6.1% in the open-repair group and 61.4%±15.2% in the EVAR group, and the 5-year survival rate was 60.8%±6.7% in the open-repair group and 61.4%±15.2% in the EVAR group. There was no significant difference between the two groups (**Fig. 1**).

## Discussion

The surgical outcomes for rAAA have improved, but mortality is still high, ranging from 20% to 30%.^10,11)^ In our study, out of the 91 patients with rAAA, 24 died in the hospital; hence, the hospital mortality rate was 26.3%.

Examining the causes of death in the 24 deaths, difficulty in hemorrhage control and postoperative intestinal necrosis accounted for the majority of the cases. Therefore, managing these two factors may significantly improve surgical outcomes.

Regarding hemorrhage control, it is important to start surgery while the patient is hemodynamically stable. In our study, among the patients who died, there were significantly more cases of preoperative shock. Seven of the ten patients who died from DIC or hemorrhage required IABO insertion or aortic clamp by thoracotomy or cardiopulmonary resuscitation. In other words, if the hemodynamics are disrupted, the surgical outcomes deteriorate.

The first touch for an rAAA was of a vascular surgeon at our hospital. In addition, EVAR was performed by a radiologist at our hospital. Therefore, even in cases where EVAR is possible, open repair may have to be selected if there is no time for the radiologist to arrive at the hospital to perform EVAR.

Recently, it has been reported that EVAR has better outcomes.^1–5)^ However, open repair have more preoperative rsks. This difference was reflected in the mortality and morbidity associated with EVAR and open repair procedures. Considering the preoperative risk, there was no difference in the long-term mortality between the EVAR and open repair; in contrast, in the high-risk group, EVAR had a higher mortality rate.^11)^ In our study, there were no deaths among the EVAR cases. Instead, all deaths occurred among the open-repair cases. Mortality was significantly higher in the open-repair group than in the EVAR group (0/13 in the EVAR group, 20/70 in the open-repair group, p=0.031). However, among the EVAR cases, there were a few cases with preoperative shock, and there were no cases requiring aortic clamping by thoracotomy or IABO insertion due to hemodynamic failure. Robinson et al.^11)^ reported that EVAR did not independently reduce long-term mortality compared to open repair. Furthermore, the time from symptom onset to incision and the time from hospital admission to incision were significantly longer in the EVAR group than in the open-repair group. In our study, there was no difference in the door-to-procedure time between the EVAR and the open-repair groups. Based on the long-term results of EVAR and open repair, there was no difference between the two groups, and thus we cannot conclude that EVAR is superior to open repair for the treatment of rAAA. The surgical procedure should be selected according to the conditions of each institution and the anatomy of the aneurysm. At our hospital, it was decided that EVAR would be performed under the supervision of a radiologist, who was also the instructor. Vascular surgeons performing EVAR alone was not permitted. Therefore, if it took a long time for the radiologist to arrive at the hospital and EVAR could not be started immediately, we had no choice but to select open repair. There were four cases of open repair due to delayed radiologist arrival. Three patients died in the hospital after surgery. However, it was unclear whether their lives could have been saved even if an immediate EVAR had been performed.

If hemodynamics are disrupted before surgery, surgery should be performed after aortic clamping by thoracotomy or IABO insertion to stabilize the hemodynamics.^12,13)^ Based on our study, the prognosis was poor if hemodynamic failure occurred. IABO is a procedure that is generally used in the trauma area,^14)^ but it should be considered as a treatment option even for rAAA. Aortic clamping by thoracotomy and IABO insertion are implemented as a last resort. It is important to start surgery before such a procedure is needed. In other words, surgery should be started before hemodynamic disruption.

Of the ten patients who required aortic clamp by thoracotomy or IABO insertion due to hemodynamic failure, two had IABO insertion and one had a left thoracotomy aortic clamp in the emergency room. In these three cases, hemodynamic failure occurred before considering the indication for EVAR, so it was necessary to transport them to the operating room and immediately open the abdomen. However, none of these patients could be saved. Preoperative hemodynamic failure is fatal and can lead to hemorrhage control difficulties during and after surgery, which then leads to death.

Regarding surgical procedures after laparotomy, it is important to avoid touching the retroperitoneum, where hematoma is detected on preoperative computed tomography scan. In the unlikely event that hematoma ruptures and blood squirts from the rupture hole due to contact with the retroperitoneum hematoma, an assistant presses the rupture hole to control hemorrhage and a surgeon immediately peels off the area near the renal artery and clamps the abdominal aorta. It was reported that the vena cava, left renal vein, left renal artery, pancreaticoduodenal vein, and spleen can be damaged by aortic clamping^12)^; therefore, caution is required. Another method for controlling hemorrhage consists of inserting an occlusion balloon into the rupture hole, but care must be taken so that there is sufficient time for balloon insertion, and hemodynamic breakdown does not occur. In addition, cutting and transecting the left renal vein expands the field of view and reduces unnecessary hemorrhage. We did not observe any complications due to transection of the left renal vein.

In the prevention of postoperative intestinal necrosis, there is a close relationship between ACS and intestinal ischemia, and morbidity and mortality increase when ACS occurs.^15–18)^ Preoperative hypotension, preoperative consciousness disorder, intraoperative massive bleeding (≥5 L), and use of IABO are considered risk factors for ACS.^18,19)^ IAP is most easily measured using bladder pressure through a urinary catheter. IAP normally ranges from 5 to 7 mmHg in critically ill patients.^20)^ Postoperative rAAA may result in an IAP ≥12 mmHg. In some cases, IAP of 12 mmHg or higher causes organ dysfunction, such as decreased renal function. In general, it has been described that when the IAP exceeds 20 mmHg, it can cause organ failure.^21)^ Paty et al.^22)^ suggested that IAP should be measured hourly and decompressive laparotomy should be performed when it exceeds 20 mmHg with end-organ dysfunction, such as reduced urinary output or ventilator difficulties with peak airway pressure.

It has been reported that OAM for ACS prevention reduces intestinal ischemia and excision and improves mortality.^7,23)^ We have also actively introduced OAM for ACS prevention in the last few years. In our study, five out of ten cases required OAM were saved and if they survived the acute phase; they were hospitalized for a long time. Acosta et al.^7)^ reported that the management of open abdomen at the time of the first surgery had a better outcome than the management of open abdomen in the second term. Even if the abdominal wall could be closed, our policy was to not force the abdominal wall to close in consideration of the risk factors of ACS mentioned above. If necessary, we actively performed OAM. It should also be noted that the diagnosis of ACS may be delayed in EVAR cases.^24)^ By avoiding ACS, intestinal ischemia/necrosis can be prevented, and the mortality rate can be reduced. For this purpose, it is necessary to perform OAM without hesitation. OAM requires long-term hospitalization and strict systemic management. In addition to infection control and nutritional management, a close relationship between postoperative management doctors and gastrointestinal surgeons is important. IAP should be measured and carefully monitored with ACS. The intestinal tract status should be monitored, and if necessary, intestinal resection and closure of the abdominal wall should be performed at an appropriate time.

**Figure 2:**
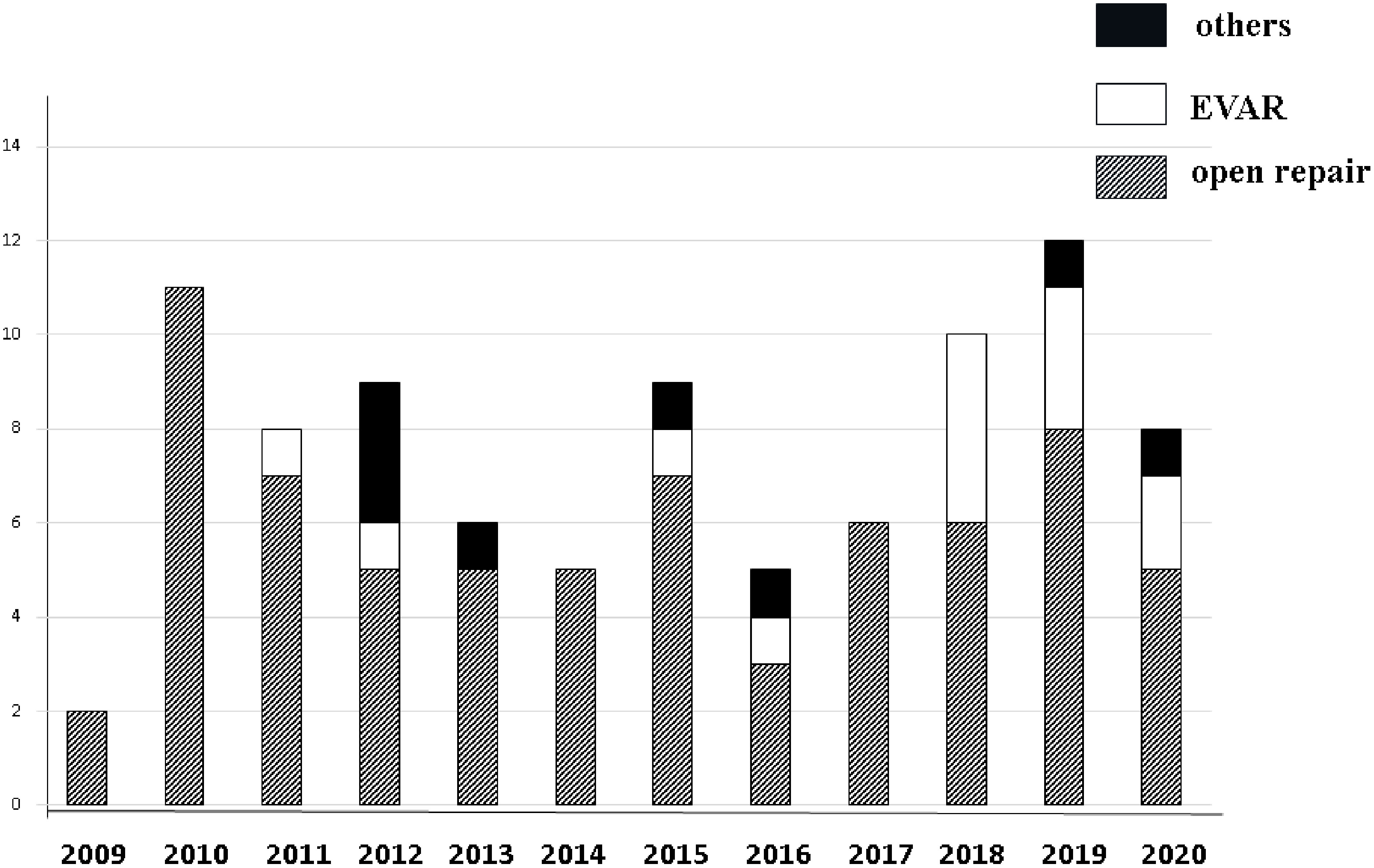
Fig. 2 Changes in the surgical procedures of ruptured abdominal aortic aneurysm according to the year at our hospital.

**Figure 2** shows the yearly changes in the surgical procedure for ruptured abdominal aortic aneurysm at our hospital. Recently, the number of full-time radiologists at our hospital has increased. Therefore, in the future, EVAR may increase as a surgical procedure for rAAA. If this happens, it is necessary to determine whether the surgical outcomes improve.

### Study limitation

In some cases, the open repair approach was selected instead of EVAR due to the absence of a radiologist. Because of this selection bias, the postoperative outcomes of the open repair and EVAR groups cannot be strictly compared. However, we compared both groups based on the current conditions of our hospital.

## Conclusion

To improve the surgical outcomes for rAAA, it is necessary to start the surgical procedure immediately when there is hemodynamic stability. Whether to select EVAR or open repair, it should be decided based on the aneurysm anatomy only, but also based on the situation of each institution. To prevent postoperative intestinal necrosis, it is necessary to consider the risk factors for ACS and actively introduce OAM.
